# Oxidative Stress and Microglial Response in Retinitis Pigmentosa

**DOI:** 10.3390/ijms21197170

**Published:** 2020-09-28

**Authors:** Yusuke Murakami, Yusaku Nakabeppu, Koh-Hei Sonoda

**Affiliations:** 1 Department of Ophthalmology, Graduate School of Medical Sciences, Kyushu University, Fukuoka 812-8582, Japan; sonodak@med.kyushu-u.ac.jp; 2 Division of Neurofunctional Genomics, Department of Immunobiology and Neuroscience, Medical Institute of Bioregulation, Kyushu University, Fukuoka 812-8582, Japan; yusaku@bioreg.kyushu-u.ac.jp

**Keywords:** retinitis pigmentosa, oxidative stress, microglia, oxidative DNA damage

## Abstract

An imbalance between the production of reactive oxygen species (ROS) and anti-oxidant capacity results in oxidative injury to cellular components and molecules, which in turn disturbs the homeostasis of cells and organs. Although retinitis pigmentosa (RP) is a hereditary disease, non-genetic biological factors including oxidative stress also modulate or contribute to the disease progression. In animal models of RP, the degenerating retina exhibits marked oxidative damage in the nucleic acids, proteins, and lipids, and anti-oxidant treatments substantially suppress photoreceptor cell death and microgliosis. Although the mechanisms by which oxidative stress mediates retinal degeneration have not been fully elucidated, our group has shown that oxidative DNA damage and its defense system are key regulators of microglial activation and photoreceptor degeneration in RP. In this review, we summarize the current evidence regarding oxidative stress in animal models and patients with RP. The clinical efficacy of anti-oxidant treatments for RP has not been fully established. Nevertheless, elucidating key biological processes that underlie oxidative damage in RP will be pivotal to understanding the pathology and developing a potent anti-oxidant strategy that targets specific cell types or molecules under oxidative stress.

## 1. Introduction

The cellular organelles and molecules of the human body are always at risk of being oxidized by reactive oxygen species (ROS), which are produced as a regular part of the body’s activity. ROS such as superoxide (^1^O_2_), hydrogen peroxide (H_2_O_2_), and hydroxyl radicals (^•^OH) are generated in the process of oxidative phosphorylation and ATP synthesis in the mitochondria [1]. Alternatively, ROS can be produced by NADPH oxidases (NOXs), which are transmembrane enzymes that form a ^1^O_2_-producing protein complex upon activation [2]. NOXs are critical for the body’s immune defense against infected bacteria as well as for health and fighting disease in a variety of tissues including the retina.

**Figure 1:**
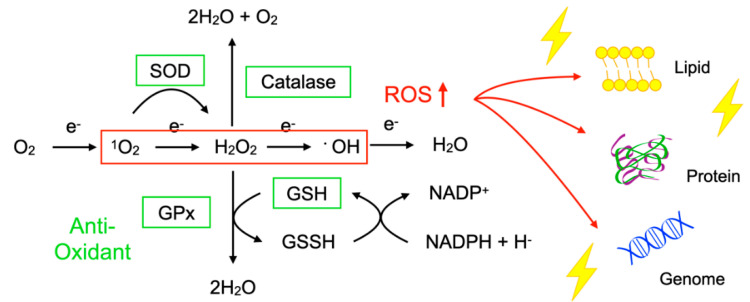
Imbalance between reactive oxygen species (ROS) and anti-oxidants impairs the function of macromolecules. ROS including superoxide (^1^O_2_), hydrogen peroxide (H_2_O_2_), and hydroxyl radicals (^•^OH) are generated during oxidative phosphorylation in the mitochondria. Superoxide dismutase (SOD) catalyzes the ^1^O_2_ into oxygen (O_2_) and H_2_O_2._ Catalase breaks down ^•^OH into O_2_ and water (H_2_O). Glutathione peroxidase (GPx) catalyzes H_2_O_2_ into H_2_O, with the conversion of glutathione (GSH) to its oxidized disulfide form (GSSG). An imbalance between ROS production (red box) and anti-oxidant capacity (green box) results in the accumulation of oxidative insults (lightning symbol) to the cellular lipids, proteins, and nucleic acids. ↑, increase.

To protect the organelles and molecules against ROS, the cells have an elaborate defense system to neutralize or catalyze ROS and repair ROS-induced damage (Figure 1). For example, superoxide dismutase (SOD) catalyzes the dismutation of ^1^O_2_ into oxygen (O_2_) and H_2_O_2_. Catalase breaks down ^•^OH into O_2_ and water (H_2_O). Glutathione peroxidase (GPx) catalyzes H_2_O_2_ into H_2_O, with the conversion of glutathione (GSH) to its oxidized disulfide form (GSSG) [3].

Oxidative stress is a state in which the balance between the production of ROS and the anti-oxidant defense system is impaired. The production of ROS is markedly increased in various disease conditions including inflammation, metabolic dysfunction, cancer, and neurodegeneration. Excessive ROS insults the cellular macromolecules such as nucleic acids, proteins, and lipids (Figure 1), leading to cellular dysfunction, transdifferentiation, or death. Accordingly, oxidative stress is generally deemed to be detrimental to human health; however, it should be noted that, in some conditions, ROS are required to mediate the body’s protection against infection and tissue injury [4].

## 2. Retinitis Pigmentosa and Oxidative Stress

### 2.1. Etiology of Retinitis Pigmentosa

Retinitis pigmentosa (RP) comprises a group of inherited retinal degeneration states that, without effective treatment, lead to blindness [5,6]. Genetic mutations associated with RP have been identified in more than 90 genes, most of which are related to the function and maintenance of rod photoreceptor cells. Rod cells are responsible for vision in dim light, and the symptoms of RP typically start with night blindness due to the dysfunction and death of rod cells. Ring scotoma at the mid-peripheral retina, which corresponds to the region containing the highest rod density, is also associated with RP. At this early stage of the disease, the daily lives and activity levels of patients are usually not severely affected. However, following rod cell loss, the remaining cone photoreceptor cells are gradually but progressively impaired, leading to constriction of the visual field and eventually the loss of central vision. This impairment of cone-mediated daylight vision is the most debilitating aspect of RP. Based on these clinical features, RP is also categorized as rod-cone dystrophy [7].

As can be seen from the variety of causal genes, RP patients show diverse heterogeneity in their phenotype. For example, the onset age of night blindness or other symptoms ranges from early childhood to the mid-30s or even later. The central visual function and its progression also have significant inter-individual variability among patients with mutations in the same gene or even among family members with the same mutation. Recent advances in genotype–phenotype technology have led to a better characterization of each causal gene in RP, but there are still significant gaps between genetic results and clinical findings [8].

Despite the heterogeneity in disease progression, there are shared clinical characteristics in RP, including the appearance of the fundus. It has been suggested that some disease-modifying factors may underlie the disease as a common etiology of RP. For example, following the rod cell loss, the degenerative retina, which largely reduces the O_2_ consumption, is exposed to higher levels of O_2_ and thereby damaged by ROS, suggesting that oxidative stress may promote rod and cone degeneration in RP [9]. Other biological factors such as inflammation, autophagy, and metabolic dysfunction have been suggested to modify the disease progression of RP [10,11,12]. Elucidation of the biological mechanisms underlying the rod and cone degeneration in RP will be important for gaining a better understanding of the disease and for the development of novel therapies targeting specific biological pathways.

### 2.2. Oxidative Damage in Animal Models of RP

#### 2.2.1. Evidence of Increased Oxidative Damage in the RP Retina

Increased ROS in the tissue can be experimentally detected by probes such as dihydroethidium (DHE) and CellROX™, which react with ROS to produce a fluorescent signal. After an in vivo administration of DHE, the red fluorescent signal can be detected in the outer retina of rd1 mice, a murine model of RP that have a mutation in *Pde6b* and a confounding mutation in *Gpr179* [13,14]. This ROS signal is not observed in the normal retina, indicating that the production of ROS is markedly increased in the degenerative loci of RP.

**Figure 2:**
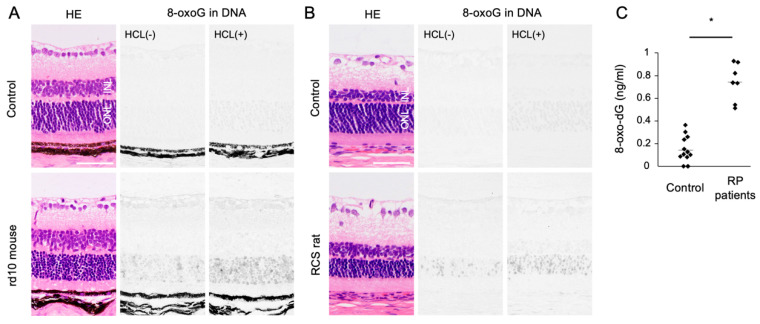
The Figure is reproduced from [16] with copyright permission. Accumulation of oxidative DNA damage in retinitis pigmentosa (RP). (**A**,**B**) Hematoxylin and eosin (HE) staining and immunohistochemical staining of 8-oxo-7,8-dihydroguannine (8-oxoG) in the retina of rd10 mice (**A**) and Royal College of Surgeons (RCS) rats (**B**), two genetically different models of retinitis pigmentosa. Per staining, HCl pretreatment was used to denature the nuclear DNA, thereby enhancing the detection of 8-oxoG in the nuclear DNA. Note that 8-oxo-G accumulation is substantially increased in the outer nuclear layer (ONL) of RP mice. Scale bar, 50 μm. INL: inner nuclear layer. (**C**) Enzyme-linked immunosorbent assay for 8-oxo-dG in the vitreous of RP patients and controls (patients with idiopathic epiretinal membrane). The vitreous levels of 8-oxo-dG are increased in RP patients. * *p* = 0.0003

Oxidized lipids, proteins, and nucleic acids can also be visualized by the specific antibodies against the oxidized residue of each molecule. For example, malondialdehyde (MDA) and 4-hydroxynonenal (4-HNE) are byproducts of lipid peroxidation; protein carbonyls and nitrotyrosine are markers of protein oxidation; and 8-oxo-7,8-dihydroguannine (8-oxoguanine, or 8-oxoG) is a major form of oxidized nucleic acids. In several models of RP, it was shown that oxidized lipids, proteins, and nucleic acids are substantially increased in the degenerative retina, especially in the photoreceptor layer (Figure 2A,B) [15,16].

In addition to the photoreceptor cells, immune cells such as microglial cells or macrophages extensively infiltrate these outer retinal regions and express oxidative molecules such as NOX2, suggesting that these inflammatory cells may be an alternative source of ROS in the RP retina [13].

#### 2.2.2. Role of Oxidative Damage in RP

Although oxidative stress has bidirectional roles (alternately beneficial and detrimental), laboratory findings suggest that oxidative stress acts to promote the retinal degeneration in RP. Yamada et al. showed that mice placed in 75% O_2_ for 2 weeks exhibited significant degeneration of photoreceptor cells, supporting the notion that a higher O_2_ supply may be deleterious to the retina [17].

In rd1 mice, Komeima et al. tested a cocktail of four anti-oxidants (vitamin E, a SOD mimetic, vitamin C, and a-lipoic acid), and they demonstrated that this anti-oxidant treatment substantially reduced the accumulation of oxidized lipids and protected cone cells against death. rd10 mice with a *Pde6b* mutation are another model of RP, and exhibit a slower progression of retinal degeneration [18]. Lee et al. treated rd10 mice with oral N-acetylcysteine (NAC), which replenishes intracellular GSH, and found that NAC substantially protected rod and cone photoreceptor cells [19]. We also assessed oral NAC for rd10 mice, and we found that NAC exerted neuroprotective as well as immunosuppressive effects [20]. The therapeutic effects of anti-oxidants have been observed in several animal models with different genetic mutations, suggesting that oxidative stress may be a common important pathology of the retinal degeneration in RP.

One limitation of these laboratory studies is that, in most of the experiments, the dosage of anti-oxidant drugs was far higher than that used in human medicine or dietary supplements. In addition, the treatment was often initiated at the very early phase in experimental models. Given that RP patients are usually referred to a hospital at the mid-phase or even late phase of the disease, the efficacy of a treatment in experimental models should be carefully interpreted.

#### 2.2.3. Modification of Anti-Oxidant Genes in RP

Gene modification to enhance the body’s anti-oxidant capacity is another approach to combat oxidative stress. Usui et al. showed that transgenic overexpressions of SOD1 and GPx4, which catalyze ^1^O_2_ and H_2_O_2_, delay the cone degeneration in rd1 mice [21]. Although transgenic overexpression is not applicable to humans, Xiong et al. demonstrated that the viral vector-mediated retinal gene transfer of an anti-oxidant gene had therapeutic potential in RP models. In rd1, rd10, and *Rhodopsin*^−/−^ mice, the adeno-associated virus (AAV) vector-mediated delivery of nuclear factor erythroid-derived 2-like 2 (NRF2), a transcription factor that boosts detoxifying and anti-oxidant genes on oxidative stimulation, is effective for cone survival [22]. Because gene therapy using AAV vector has been approved for Leber’s congenital amaurosis and has been widely tested in clinical trials for inherited retinal degeneration and other retinal diseases, local and long-lasting anti-oxidant therapy may be an alternative strategy for chronic retinal degenerative disorders including RP [23,24].

### 2.3. Clinical Evidence of Oxidative Stress in RP

Since human retinal samples are rarely obtained with an immediate sample preparation to prevent post-mortem oxidation, oxidative stress in RP patients has been analyzed using the aqueous humor, vitreous body, and peripheral blood samples. In ocular samples, we showed that 8-oxo-7,8-dyhydro-2′-deoxyguanosine (8-oxo-dG), a marker of oxidative DNA damage, is increased more than 5-fold in the vitreous of RP patients compared to controls without retinal degeneration (Figure 2C) [16]. Consistent with this finding, Campochiaro et al. demonstrated an approximately 2-fold increase of protein carbonyl contents in the aqueous humor of RP patients [25]. On the other hand, anti-oxidant molecules such as GSH and SOD3 were decreased in the aqueous humor of RP patients [26]. These findings suggest that an oxidative imbalance occurs in the eyes of RP patients, which is consistent with findings in experimental models.

The oxidant and anti-oxidant profiles in the peripheral blood of RP patients have shown some conflicting results. Martínez-Fernández de la Cámara et al. reported increased oxidative markers (e.g., nitrotyrosine and thiobarbituric acid reactive substances) but decreased anti-oxidant SOD3 in RP patients [26]. In contrast, Campochiaro et al. demonstrated no differences in protein carbonyls, the GSH/GSSH ratio, or SOD3 in the serum of RP patients [25]. In our cohort of 52 RP patients, we investigated an oxidant marker (hexanoyl-lysine (HEL)) and three anti-oxidant markers (SOD3, GPx, and potential anti-oxidant (PAO)). We observed no significant difference in these four markers between RP patients and healthy controls; however, a subgroup analysis showed that the serum SOD3 activity was significantly lower in the RP patients with severe degeneration involving the macula [27]. In addition, the lower serum SOD3 activity in the RP patients was related to worse visual acuity and macular retinal sensitivity [27]. These data suggest that the decline of serum SOD3 activity is associated with the loss of cone-mediated central vision.

There are two possible interpretations for the lower serum SOD3 in patients with advanced RP. One possibility is that the serum SOD3 level falls in response to retinal degeneration and increased ocular ROS in RP. Another possibility is that RP patients with a lower baseline anti-oxidant capacity have a faster disease progression. This point should be addressed in future studies by directly comparing oxidant/anti-oxidant molecules between aqueous and serum samples as well as by following up the patients to determine the longitudinal changes of the serum SOD3 activity and central vision.

## 3. Clinical Outcomes of Anti-oxidant Treatments

### 3.1. Anti-oxidant Supplements for Systemic Diseases

Vitamins and minerals have potent anti-oxidant ability, and they are commonly taken as dietary supplements to promote good health. Vitamin E is located within the phospholipid layer of the cell membrane and prevents lipid peroxidation. Vitamin C can restore the oxidized vitamin E to the reduced form. Carotenoids (e.g., β-carotene, lutein, zeaxanthin) and polyphenols (e.g., anthocyanidins, catechins, resveratrol) scavenge or reduce the ROS in a direct or indirect manner [28].

The Physicians’ Health Study II (PHS-II) and the SUpplementation in VItamins and Mineral AntioXidants Study (SU.VI.MAX) are two large trials investigating the health outcomes afforded by multivitamin supplements. The PHS-II study included 14,641 male U.S. physicians, and a multivitamin containing 30 formulations was used as an intervention for a 11.2-year follow-up period. The SU.VI.MAX study was conducted in 13,017 French individuals of both sexes with a 7.5-year follow-up, and an intervention group was supplemented with a five-ingredient multivitamin (vitamin C, vitamin E, β-carotene, selenium, and zinc). Both of these studies reported a lower cancer incidence in the men who were taking the multivitamin supplements, whereas no difference in the incidence was revealed in the women [29,30]. This benefit in men was relatively small with borderline significance, and a meta-analysis including more than 100,000 individuals did not show any effects on cancer incidence from the multivitamins. In addition, there were no significant differences in cardiovascular disease (CVD) and mortality due to anti-oxidant supplements [31]. The U.S. Preventive Services Task Force (USPSTF) concluded that there is insufficient evidence to recommend for or against the use of anti-oxidant supplements to prevent cardiovascular disease or cancer.

In addition, β-carotene has been shown to increase the risk of lung cancer in individuals who smoke tobacco or have an exposure to asbestos. This was shown in two large trials [32,33] and confirmed in a meta-analysis with an odds ratio of 1.24 in current smokers [34]. These findings suggest that anti-oxidants may not necessarily be beneficial, as expected from the diverse biological functions of ROS.

### 3.2. Anti-oxidant Supplements for Ocular Disorders

#### 3.2.1. Anti-oxidant Supplements for Age-related Macular Degeneration (AMD)

The Age-Related Eye Disease Study (AREDS) investigated the clinical course of age-related macular degeneration (AMD) and cataracts and assessed the impact of anti-oxidants on these eye diseases. For the AMD trial, 3640 participants with AMD regions in the fundus were randomly divided into the following four treatment arms: (1) antioxidants (vitamin C, vitamin E, and β-carotene), (2) zinc, (3) anti-oxidants and zinc, or (4) placebo. In a mean 6.3-year follow-up, the data showed that the use of anti-oxidants plus zinc reduced the risk of developing advanced AMD, with an odds ratio of 0.72 [35]. 

The AREDS2 assessed whether the addition of lutein/zeaxanthin and/or omega-3 fatty acids to the original AREDS formulation further reduces the risk of AMD progression. For this study, 4203 participants were primarily randomized to the following four treatment arms: (1) lutein/zeaxanthin, (2) omega-3 fatty acids, (3) lutein/zeaxanthin plus omega-3 fatty acids, or (4) placebo. Among the participants, 3036 agreed to the secondary randomization of the AREDS formulation into the following four categories: (1) the original AREDS formulation (vitamin C, vitamin E, β-carotene, and zinc), (2) the AREDS formulation without β-carotene, (3) the AREDS formulation with a lower zinc dose, and (4) the AREDS formulation with no β-carotene and a lower zinc dose. In a mean 5-year follow-up, lutein/zeaxanthin and/or omega-3 fatty acids did not show a clear benefit against AMD progression. However, a secondary analysis of the results demonstrated that the lutein/zeaxanthin-containing AREDS supplements reduced the risk of AMD progression (odds ratio: 0.82) compared to the β-carotene-containing AREDS supplements. Given the potential risk for increasing the incidence of lung cancer, the AREDS Study Group recommended that lutein/zeaxanthin could be a safer carotenoid substitute for β-carotene in the AREDS formulation [36].

The PHS-II study also evaluated the incidence of AMD as well as those of cardiovascular diseases and cancer; 14,236 male U.S. physicians who did not have a diagnosis of AMD at baseline were randomized to treatment groups with a multivitamin, vitamin C, vitamin E, and a placebo. After an 8-year follow-up, the results indicated that neither vitamin C nor vitamin E had significant beneficial or harmful effects on the incidence of AMD [37]. The multivitamin supplement also did not show any benefit or harm regarding the risk of AMD in a mean 11.2-year follow-up [38]. The subjects included in the PHS-II study were associated with a lower risk of AMD compared to the AREDS subjects, and there appears to be insufficient evidence to conclude that an anti-oxidant supplement can prevent AMD in general populations without high-risk profiles.

#### 3.2.2. Anti-oxidant Supplements for RP

Because RP is a relatively rare disease, a large randomized trial to assess the effects of anti-oxidant supplements may not be practical. Vitamin A has been traditionally used for RP, based on the results of a randomized trial showing that vitamin A slowed down the decline of a cone-derived 30-Hz ERG response among 601 subjects with RP. However, it should be noted that there were no significant changes in the rate of the decline of the visual field or visual acuity [39]. 

Berson et al. conducted a post hoc analysis assessing whether omega-3 intake could affect the visual outcome among 357 RP patients who received daily vitamin A in three clinical trials [40]. A slower rate of decline of visual acuity was observed in the patients with high omega-3 intake, whereas there was no significant difference in the cone ERG response between the patients with a high or low omega-3 intake [40].

In another trial, Hoffman et al. addressed whether docosahexaenoic acid (DHA), a major omega-3 fatty acid, could slow the progression of X-linked RP (the DHAX Trial); 78 patients were randomized to treatment with (1) DHA and a multivitamin or (2) the multivitamin alone [41]. The cone ERG response, the primary outcome of that trial, was not significantly different between the groups [41]. However, in ancillary outcome analyses, the rate of the decline of visual field sensitivity was reduced with the DHA supplementation [42].

There have been additional trials assessing the effects of lutein or β-carotene on the progression of RP, and their outcomes are not consistent with respect to the ERG response, visual acuity, or retinal sensitivity, as in the trials described above [43,44].

Given the lack of effects on the incidence of cancer or cardiovascular diseases and the modest impact on AMD progression in a limited population, establishing clear evidence of the merits and demerits of supplement therapy for RP may require larger sample sizes and longer follow-ups. The results of clinical trials of vitamins and minerals for systemic and eye diseases are summarized in Table 1 and Table 2. Note that this review focuses mainly on retinal degeneration and oxidative stress, and we did not introduce all of the trials assessing anti-oxidant supplements.

### 3.3. Oral NAC Treatments for RP

Campochiaro et al. recently conducted a Phase 1 clinical trial of the anti-oxidant NAC for RP patients, based on its safety and efficacy in RP model mice [45]. Thirty RP patients were subjected to a dose escalation study with 600 mg (cohort 1), 1200 mg (cohort 2), and 1800 mg NAC (cohort 3) 2×/day for 12 weeks and thereafter 3×/day for 12 weeks. Visual acuity was significantly improved by 0.4, 0.5, and 0.2 letters/month in cohorts 1, 2, and 3, respectively. Retinal sensitivity in the macula was also improved by 0.15 dB/month in cohort 3. The drug-related gastrointestinal adverse events were mild and resolved spontaneously [45]. The efficacy of NAC for RP patients should be addressed in a future trial involving more patients.

## 4. Oxidative DNA Damage and its Repair System

### 4.1. Mechanisms by Which Oxidative Stress Mediates Retinal Degeneration

Although the biological actions of anti-oxidant supplements are evident, their clinical benefits have been relatively modest or unclear. Does this mean oxidative stress does not have a major role in these diseases? Accumulated evidence from experimental and clinical studies supports the role of oxidative stress in various disorders including RP. Alternatively, oral anti-oxidant supplements may not be sufficient to detoxify excessive ROS in the diseased loci. In this scenario, a stronger inactivation of ROS would be required; however, because ROS mediate both pro-survival and detrimental functions, an indiscriminate suppression of oxidative stress may not be feasible for human health. An ideal approach could therefore be the targeting of harmful oxidation in the diseased loci or within the specific cellular compartment, with enhanced efficacy and a reduction of adverse effects.

However, the mechanisms by which oxidative stress mediates retinal degeneration remain largely unexplored. In the sections below, we focus on oxidative DNA damage and its related signaling as a mechanism of harmful oxidation. Other oxidative insults such as those on proteins and lipids may also contribute to the harmful effects, but a comprehensive coverage of oxidative insult mechanisms is beyond the scope of this review and can be found in other excellent reviews [46,47,48].

### 4.2. DNA Oxidation and Mismatched Base Pair Formation

DNA carries the genetic information that produces the RNA and proteins required for cellular homeostasis and life itself. Oxidative damage to nucleic acids is thus hazardous because it can cause significant alterations or the destruction of gene structures. Guanine is the most susceptible nucleobase, and 8-oxoG is frequently used as a marker of oxidative DNA damage. There are two sources of oxidized DNA—one is from the direct oxidation of genomic DNA by ROS and the other is from the incorporation of oxidized bases in the nucleotide pool. It has been shown that guanine in the nucleotide pools tends to be more oxidized than that in the genomic DNA [49,50]. During DNA replication, 8-oxoG can mispair with adenine, resulting in the formation of somatic mutations [49,50].

### 4.3. Counter-Mechanisms against Oxidative DNA Damage

**Figure 3:**
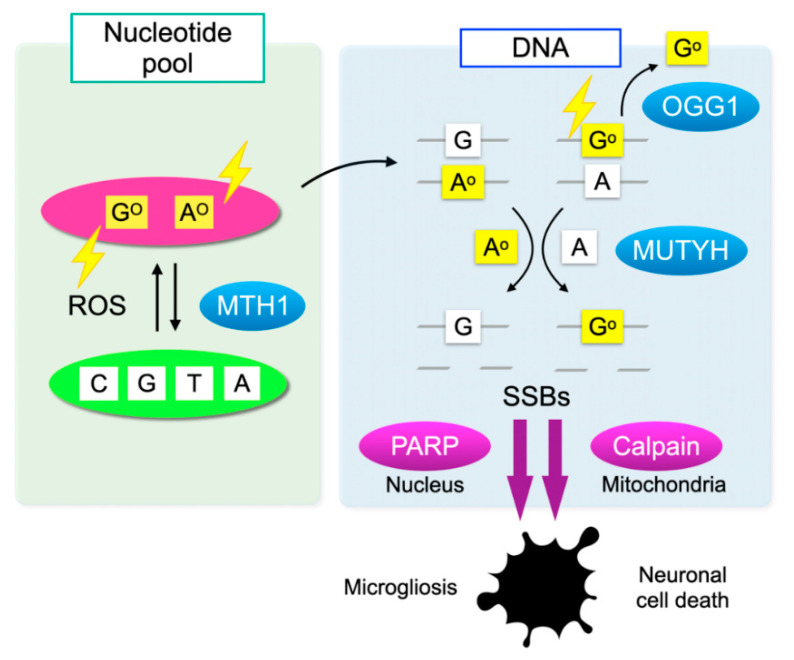
Oxidative DNA damage and its repair system. Oxidation of DNA occurs via two processes: one is the direct oxidation by reactive oxygen species (ROS) and the other is the incorporation of oxidized bases from the nucleotide pool. Oxidative insults are shown in lightning symbols. During DNA replication, 8-oxoG can mispair with adenine, resulting in the formation of somatic mutations. Mut T homolog-1 (MTH1) hydrolyzes oxidized purine nucleoside triphosphates, such as 8-oxo-7,8-dihydrro-2′-deoxyguanosine 5′-triphosphate (8-oxo-dGTP) and 1,2-dihydro-2-oxo-2′-deoxyadenosine 5′-triphosphate (2-oxo-dATP) in the nucleotide pool, thereby preventing the incorporation of oxidized nucleotides into DNA. On the one hand, 8-oxoG in DNA is excised by 8-oxoguanine DNA glycosylase-1 (OGG1). On the other hand, the Mut Y homolog (MUTYH) excises the mismatched adenine opposite 8-oxoG, and 2-oxoA opposite guanine. MUTYH can suppress mutagenesis caused by oxidative DNA damage; however, under severe oxidative DNA damage, MUTYH-mediated BER leads to accumulation of single-strand breaks (SSBs) in DNA. SSBs in the nuclear DNA activates poly(ADP-ribose) polymerase (PARP) and SSBs in mitochondrial DNA results in calpain activation through mitochondrial dysfunction, which in turn induces microgliosis and neuronal cell death during neurodegeneration, respectively.

Cells have defense mechanisms to counter oxidative DNA damage. MutT homolog-1 (MTH1), also known as nudix hydrolase 1 (NUDT1), hydrolyzes oxidized purine nucleoside triphosphates, such as 8-oxo-7,8-dihydrro-2′-deoxyguanosine 5′-triphosphate (8-oxo-dGTP) and 1,2-dihydro-2-oxo-2′-deoxyadenosine 5′-triphosphate (2-oxo-dATP) to the monophosphate forms. MTH1 sanitizes the oxidized nucleotides in the nucleotide pool, thereby preventing their incorporation into DNA (Figure 3) [51].

On the one hand, 8-oxoG in DNA is excised by 8-oxoguanine DNA glycosylase-1 (OGG1) and then replaced with guanine through base excision repair (BER). On the other hand, the Mut Y homolog (MUTYH) excises the mismatched adenine opposite 8-oxoG, and 1,2-dihydro-2-oxo-adenine (2-oxoA) opposite guanine. MUTYH-mediated BER can repair the somatic mutations induced by oxidative DNA damage; however, under severe oxidative DNA damage, the excessive activation of MUTYH leads to the formation of single-strand breaks (SSBs) of DNA, leading to disturbed homeostasis and cell death (Figure 3) [52].

Deficiency in *Mth1*, *Ogg1*, or *Mutyh* in mice increases the spontaneous mutation frequency in DNA, resulting in the increased incidence of cancer [53]. In neurodegenerative conditions, MTH1 and OGG1 exhibit a neuroprotective function by preventing the accumulation of 8-oxoG. The neurodegeneration induced by 3-NP is markedly enhanced in *Mth1*/*Ogg1* double-knockout mice, accompanied by an increase in 8-oxoG accumulation in the degenerating neurons and the activated microglia [54]. In contrast, MUTYH promotes neurodegeneration under oxidative stress. As described above, adenine excision by MUTYH forms SSBs in the nuclear and mitochondrial DNA, which activates the cellular stress response [52]. Neuronal cell loss and microgliosis are attenuated in *Mutyh* ^−/−^ mice in the 3-NP neurodegeneration model [54].

## 5. DNA Oxidation in Microglia Promotes Inflammation and Degeneration in RP

### 5.1. Accumulation of 8-oxoG in RP

An accumulation of oxidative DNA damage is associated with neurodegenerative diseases including Alzheimer’s disease and Parkinson’s disease [55,56]. In animal models of RP, we previously showed that 8-oxoG accumulation is substantially increased in the outer nuclear layer (ONL) where photoreceptor cells reside. These changes were consistently observed in two RP models with different genetic mutations (Figure 2A,B). In addition, vitreous samples from RP patients contained higher levels of free 8-oxo-dG compared to control subjects without retinal degeneration (Figure 2C) [16]. There findings suggest that oxidative DNA damage may underlie RP as a common pathology.

### 5.2. Role of Oxidative DNA Damage in RP

**Figure 4:**
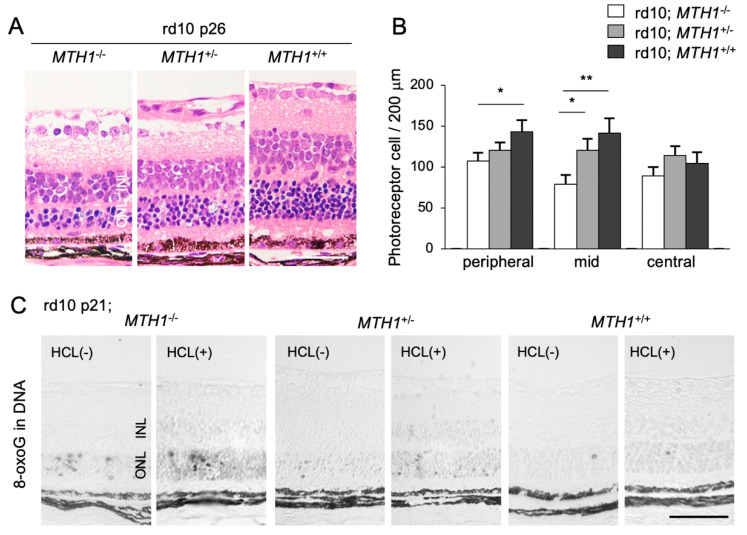
The Figure is reproduced from [16] with copyright permission. Transgenic overexpression of human *Mut T homolog-1* attenuates oxidative DNA damage and suppresses retinal degeneration. (**A**) Histological examination of rd10 mice, rd10 mice with hemizygous human *MutT homolog-1* (*hMTH1*) expression (rd10; *hMTH1-Tg*^+/−^), or rd10 mice with homozygous *hMTH1* expression (rd10; *hMTH1-Tg*^+/+^). Scale bar, 50 μm. INL: inner nuclear layer. ONL: outer nuclear layer. (**B**) Quantitative analysis of the number of photoreceptor cells in the ONL. Note that transgenic overexpression of *hMTH1* significantly suppresses photoreceptor cell loss in rd10 mice. (**C**) Immunohistochemical staining of 8-oxoG in the retina of rd10, rd10; *hMTH1-Tg*^+/−^, or rd10; *hMTH1-Tg*^+/+^ mice. 8-oxo-G accumulation in rd10 mice is attenuated by transgenic *hMTH1* expression. Scale bar, 50 μm. * *p* < 0.05. ** *p* < 0.01.

To elucidate the role of oxidative DNA damage in RP, we assessed the effect of a transgenic overexpression of human *MTH1* (*hMTH1*) on retinal degeneration [16]. The accumulation of 8-oxoG in the rd10 mouse retina is attenuated by *hMTH1* overexpression, which in turn leads to a suppression of photoreceptor cell death (Figure 4A–C) [16]. These findings suggest that a large proportion of oxidative DNA damage in the ONL of rd10 mice is derived from the oxidized nucleotide pool. This process (i.e., the incorporation of oxidized bases into the nuclear DNA) has been traditionally thought to occur during DNA replication in cell division [49]. However, because photoreceptor cells are post-mitotic neuronal cells, it is less likely that photoreceptors re-enter the cell cycle and replicate their DNA. Alternatively, other non-neuronal proliferating cells in the ONL, such as microglia, may incorporate oxidized nucleic acids into their nuclear DNA during retinal degeneration.

### 5.3. Oxidative DNA Damage Mediates Microglial Activation through MUTYH-mediated SSB Formation, Thereby Promoting Retinal Degeneration

**Figure 5:**
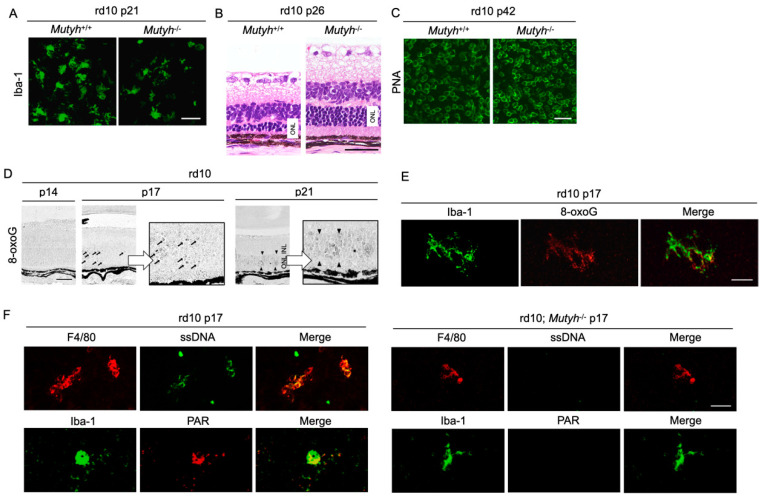
The Figure is reproduced from [59] with copyright permission. Oxidative DNA damage in microglia mediates retinal inflammation and degeneration through Mut Y homolog activation. (**A**–**C**) Immunohistochemical staining of microglial marker Iba-1 (**A**), hematoxylin and eosin staining (**B**), and peanut agglutin (PNA) staining, which labels the cone inner and outer segments (**C**) in rd10 mice or rd10 mice deficient for *Mut Y homolog* (*Mutyh*) (rd10; *Mutyh*^−/−^). Note that microgliosis and rod as well as cone degeneration are suppressed by *Mutyh* deficiency. ONL: outer nuclear layer. Scale bar, 50 μm (**A**,**B**), 20 μm (**C**,**D**). Time-dependent changes of oxidative DNA damage in rd10 mice. Scattered 8-oxoG accumulation is observed at early phase of retinal degeneration (postnatal day 17: P17), and thereafter 8-oxo-G accumulation expands to the ONL diffusely at P21 in rd10 mice. Scale bar, 50 μm. (**E**) Part of scattered 8-oxo-G staining in the P17 rd10 retinas are colocalized with Iba-1-positive microglial cells, suggesting that 8-oxo-G accumulation in microglia precedes the peak of photoreceptor cell death in rd10 mice. Scale bar, 20 μm. (**F**) Immunohistochemical staining of single-strand DNA (ssDNA) to detect single-strand breaks (SSBs) and poly(ADP-ribose) (PAR) for the marker of poly(ADP-ribose) polymerase (PARP) activation in rd10 or rd10; *Mutyh*^−/−^ mice. Microglia in rd10 mice are associated with SSB formation and PARP activation, which is reversed by *Mutyh* deficiency. Scale bar, 20 μm.

To further determine the mechanisms by which oxidative DNA damage promotes retinal degeneration, we employed rd10 mice to assess the function of MUTYH, which excises adenine mispaired with 8-oxoG [57]. As described above, previous studies demonstrated that MUTYH deficiency prevents SSB formation and cell death under oxidative stress [58,59]. Consistently, our study showed that MUTYH deficiency substantially suppresses the formation of SSBs and attenuates rod and cone photoreceptor cell death as well as microgliosis in rd10 mice (Figure 5A–C) [57]. These findings suggest that MUTYH-mediated BER is critical to promote retinal degeneration and inflammation in RP.

Neuronal cells and microglia closely interact during neurodegeneration [60], and it has not been determined which cells are the primary target of oxidative stress. Our analysis of time-dependent changes of oxidative DNA damage in rd10 mice showed that a nuclear accumulation of 8-oxoG in microglia occurs before the peak of photoreceptor degeneration, and thereafter the 8-oxoG accumulation expands to the photoreceptor nuclei along with microglial activation (Figure 5D,E) [57]. These findings suggest that the microglia may be a key target of oxidative stress in RP, and oxidative microglial activation may trigger the vicious cycle of non-resolved neuroinflammation and degeneration in RP. In accord with this idea, anti-oxidant treatments have been shown to robustly suppress the microglial activation and prevent the production of pro-inflammatory molecules in models of retinal degeneration [20,61].

Oka et al. demonstrated that SSBs formed by MUTYH-mediated BER trigger two distinct pathways—nuclear SSBs induce poly(ADP-ribose) polymerase (PARP) activation and mitochondrial SSBs mediate calpain activation [58]. Consistent with these findings, we observed that nuclear DNA oxidation in the microglia of rd10 mice is associated with PARP activation, and this PARP activation is reversed by MUTYH deficiency (Figure 5F) [57]. In addition, systemic treatment with a PARP inhibitor dampens retinal degeneration in rd10 mice, suggesting that PARP activation in microglia may be an alternative target in RP [16]. Because systemic and long-term PARP inhibition may be associated with non-negligible adverse events, the selective targeting of retinal oxidized microglia may be an attractive strategy to combat detrimental oxidative stress in RP and neurodegeneration.

## 6. Conclusions

Although RP is a hereditary disease, non-genetic environmental factors such as oxidative stress modulate the disease progression. Oxidative damage is markedly increased in the outer retina of animal RP models as well as in the ocular samples of RP patients. Anti-oxidant interventions, by either pharmacological or genetic approaches, substantially delay photoreceptor cell death in experimental RP, suggesting that oxidative stress is a key contributor to retinal degeneration in RP.

Contrary to these experimental findings, the clinical evidence that anti-oxidant supplements retard the progression of RP has not been fully established. Because anti-oxidant agents are frequently used at extremely high doses in experimental models, stronger ROS inactivation may be required to achieve clinical efficacy in RP patients. To avoid the systemic adverse effects due to long-term anti-oxidant treatment, the targeting of harmful oxidation in the retina would be a more feasible approach. To realize this strategy, a further clarification of the mechanisms by which oxidative stress mediates retinal degeneration in RP is necessary. For example, our research revealed an intimate link between oxidative DNA damage and neuroinflammation, and we identified a MUTYH-SSBs-PARP pathway in microglial cells as a therapeutic target for RP. The elucidation of the key biological processes that underlie oxidative retinal damage will lead to a better understanding of the disease and to the development of novel anti-oxidant therapies for RP.

## Figures and Tables

**Table 1:** Treatment outcomes of anti-oxidant supplements in cardiovascular disease (CVD), cancer, and age-related macular degeneration (AMD).

Study	Study Design and Supplements	Years of Follow-Up	n	Outcome	Reference
PHS-II	2 × 2 × 2 × 2 RCT, b-carotene, vitamin-E, vitamin-C, multivitamin or placebo	11.2	14,641	CVD ↔	[29]
				Prostate cancer ↓ with multivitamin	
				Other cancer ↔	
				Mortality ↔	
				AMD ↔	
SU.VI.MAX	2-arm RCT, Multivitamin or placebo	7.5	13,017	CVD ↔	[30]
				Cancer ↔ (in men ↓)	
				Mortality ↔ (in men ↓)	
Fortmann et al.	Systematic review	NA	324,653	CVD ↔	[31]
				Cancer ↔	
				Mortality ↔	
				Lung cancer ↑ with b-carotene in current smokers	
				
AREDS	4-arm RCT, (1) antioxidants (vitamin-C, vitamin-E and b-carotene), (2) Zinc, (3) antioxidants and zinc, or (4) placebo.	6.3	3640	Development of advanced AMD ↓ in subjects with AMD regions	[35]
	
				
AREDS2	4-arm RCT, (1) lutein/zeaxanthin, (2) omega-3 fatty acid, (3) lutein/zeaxanthin plus omega-3 fatty acid or (4) placebo in addition to AREDS formulation (AREDS-F)	5.0	4203	Development of advanced AMD ↔ with addition of lutein/zeaxanthin and/or omega-3 fatty acid	[36]
	
			
				
	Secondary randomization, (1) AREDS-F, (2) AREDS-F without b-carotene, (3) AREDS-F with lower zinc, or (4) AREDS-F with no b-carotene and lower zinc			Development of advanced AMD ↓ with AREDS-F plus lutein/zeaxanthin compared with b-carotene containing AREDS-F	
			
			
			

↓, decrease; ↑, increase; ↔ no difference.

**Table 2:** Treatment outcomes of anti-oxidant supplements in retinitis pigmentosa.

Study	Study Design and Supplements	Years of Follow-Up	n	Outcome	References
Berson et al.	2 × 2 RCT, vitamin A, vitamin E andVitamine A plus vitamin E or placebo	5.2	601	Cone ERG response ↑ with vitamin A	[39]
				Visual acuity and field loss ↔	
Berson et al.	post hoc analysis of omega-3 intake in 3 clnical trials which had tested vitamin A	4~6	357	Rate of visual acuity loss ↓ with high omega-3 intake	[40]
			
				Cone ERG response ↔	
DHAX	2 arm RCT, DHA or placebo	4.0	78	Cone ERG response ↔	[41,42]
	* all participants received multivitamin			Rate of visual sensitivity loss ↓ with DHA	
Berson et al.	2 arm RCT, Lutein or placebo	4.0	225	Rate of visual sensitivity loss ↔	[43]
	* all participants received vitamin A			Cone ERG response	
Rotenstreich et al.	2 arm RCT, 9-cis b-carotene or placebo, crossover	0.7	34	Cone ERG response ↑ with 9-cis b-carotene	[44]
			
				Visual acuity and field area ↔	
Campochiaro et al.	Phase 1, 3 dose escalation of oral NAC	0.5	30	Visula acuity ↑ with all dosage	[45]
				Visual sensitivity ↑ with high dosage	
				Ellipsoid zone width ↔	

↓, decrease; ↑, increase; ↔ no difference.
